# How a salt pan ant *Cataglyphis fortis* navigates artificially complex environments

**DOI:** 10.1242/jeb.249369

**Published:** 2025-02-20

**Authors:** Marilia Freire, Morgan M. Oberweiser, Antonio Bollig, Grit Kunert, Markus Knaden

**Affiliations:** ^1^ Max Planck Institute for Chemical Ecology, Department of Evolutionary Neuroethology, Hans-Knoell-Strasse 8, 07745, Jena, Germany; ^2^ Zoological Institute and Museum, General and Systematic Zoology, Loitzer Strasse 26, 17489, Greifswald, Germany; ^3^ Max Planck Institute for Chemical Ecology, Department of Biochemistry, Hans-Knoell-Strasse 8, 07745, Jena, Germany

**Keywords:** *Cataglyphis*, Idiosyncrasy, Navigation, Foraging, Homing

## Abstract

The desert ant *Cataglyphis fortis* inhabits the harsh and featureless North African saltpans. Individuals forage long distances and return to their inconspicuous nest entrance using path integration, but also rely on visual and olfactory landmarks. Here, we investigated the navigational decision making of these ants in differently structured environments. While individual ants show consistent route preferences, significant variability exists between individuals. Furthermore, the ants favor repetitive routes, suggesting that vision-based learning mechanisms and motor responses guide their navigation, with similar visual cues leading to similar egocentric decisions. This formation of idiosyncratic routes, seen in other ant species, appears to be conserved in *C. fortis* despite its usually flat habitat.

## Teaser

**Summary:** The desert ant *Cataglyphis fortis* is adapted to the rather featureless saltpans of Tunisia. However, when facing artificially complex environments, the ants exhibit navigational strategies that are surprisingly similar to those of ants that evolved within complex habitats.

## INTRODUCTION

Desert habitats impose ecological challenges, demanding adaptations in resident species such as *Cataglyphis fortis*. This ant species has developed morphological (e.g. elongated limbs for rapid locomotion), physiological (e.g. thermophilic traits) and cognitive adaptations to navigate the arid, exposed landscapes of North African saltpans (Wehner, 2020). Efficient foraging and homing strategies, supported by robust memory (Huber and Knaden, 2018) and neuronal plasticity (Rössler, 2019), are fundamental for its survival.

*Cataglyphis fortis* ants forage individually for dead arthropods in the featureless saltpans. Probably because of the high ground temperatures inhibiting chemical trails and the uneven distribution of food, the ants do not use trail pheromones for navigation (Dietrich and Wehner, 2003; Wehner, 2003) as other ants do (Barrie et al., 2023). Instead, they rely on path integration (PI), using a ‘sky compass’ for direction and step integration for distance (Knaden and Graham, 2016; Müller and Wehner, 1988; Wittlinger et al., 2006). Despite the inhospitable environment, *C. fortis* can also use environmental cues, including visual (Knaden and Wehner, 2005), olfactory (Steck et al., 2009), and tactile (Seidl and Wehner, 2006) stimuli, with visual cues best studied because of the experimental practicality of working with visual cues (Zeil, 2012).

Understanding the navigation strategies of *C. fortis* in featureless environments has provided valuable insights into the complexity of ant navigation. *Cataglyphis fortis* can develop distinctive routes even when introduced to artificial landmarks, emphasizing their cognitive processing of spatial information (Buehlmann et al., 2015; Collett, 2010). Studies confirm that *C. fortis* foragers follow individual-specific routes without added landmarks, and GPS tracking has revealed distinct initial heading directions (Buehlmann et al., 2015). The consistency of these routes, similar to behaviors in *Melophorus bagoti* and *Cataglyphis velox*, suggests a well-developed navigational strategy (Kohler and Wehner, 2005; Mangan and Webb, 2012; Schwarz and Cheng, 2010). Research on *M. bagoti* shows how ants navigate obstacle courses using views, landmarks and routes, demonstrating their adaptability to structured environments (Wystrach et al., 2011a). These studies exemplify how ants incorporate visual landmarks and panoramic views into their navigation, shaping their foraging routes.

Building on this background, our study investigated how *C. fortis* adapts its navigational strategies to structured landscapes. We focused on interactions with artificial landmarks to assess the flexibility of the ants' navigation system and to explore the cognitive processes guiding decision making in altered terrains. To do this, we introduced artificial landmarks with varying complexity and configuration to measure their impact on route formation and modification.

## MATERIALS AND METHODS

### Ants

The study subjects [desert ant *Cataglyphis fortis* (Forel 1902)] are native to a Tunisian salt pan near Menzel Chaker (Sebkhet Bou Jemel, coordinates: 34°96′N, 10°41′E). Experiments were conducted in May and June of 2021 and 2022, testing only female foragers.

### Idiosyncrasy in an artificially complex environment

#### Experimental design

**Fig. 1.**
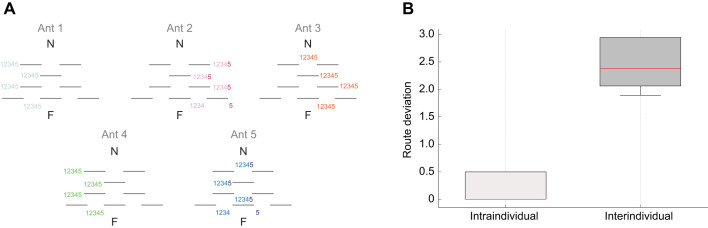
**Idiosyncrasy in a complex environment.** (A) Each diagram depicts where an individual ant has passed the maze along five consecutive natural homing runs (1–5), where N refers to the nest and F to the artificial feeder. Diagrams not drawn to scale. (B) Intraindividual and interindividual route fidelity; boxplots depict intraindividual and interindividual standard deviations (see Materials and Methods; red line, median; box, interquartile range; whiskers, full range) (paired *t*-test; *t*=−7.697, d.f.=4, *P*=0.002).

Eight metal barriers, each 15 cm high and 100 cm long, were placed at varying intervals between a *C. fortis* colony and a feeding site (i.e. an artificial depression in the ground filled with biscuit crumbs situated 10 m away from the nest). These barriers were arranged perpendicularly to the nest-feeder direction, forming a maze with multiple potential routes (Fig. 1A). After several hours of training, the movement of individually color-marked ants through this maze was monitored over five consecutive natural homing runs per ant from the feeding site to the nest, with their navigational choices systematically recorded. To do this, we focused on which passage points between the barriers were chosen by the ants.

#### Quantification and statistical analysis

The analytical approach aimed to quantify the variability in the paths chosen by individual ants, focusing on two types: intraindividual and interindividual variability. Intraindividual variability assessed how much a single ant's route varied across different trials. The first trial's path was used as a reference, and deviations in subsequent paths were recorded at each obstacle row in the maze. A scoring system was applied: no deviation from the reference path scored 0, a deviation to the nearest passage point scored 1, and further deviations scored incrementally higher based on the distance from the reference path. The total score from each path was calculated, resulting in four scores (one ‘reference run’ compared with four subsequent runs) per ant. The standard deviation of those scores was used as a measure of intraindividual variability.

Interindividual variability measured differences between the paths chosen by different ants. For each ant, the most frequently chosen path across trials was determined and compared with the paths of other ants, using the same scoring system as for intraindividual variability. The standard deviation of each ant for these scores provided a measure for interindividual variability. A paired *t*-test was used to statistically compare the standard deviations of intraindividual and interindividual variability with data normality confirmed via a quantile–quantile plot (qqPlot).

### Rules governing decision making along complex paths

#### Experimental design

**Fig. 2.**
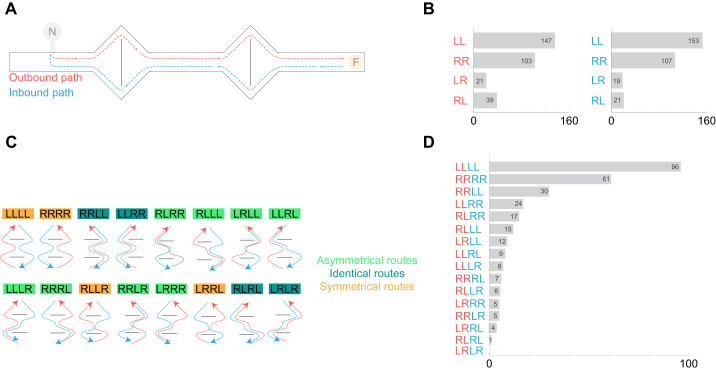
**Decision-making dynamics in structured environments.** (A) The maze layout used in the experiments; dashed lines are example routes an ant might take along its outbound (red) and inbound (blue) path (N, nest; F, feeder). (B) Frequency of each possible outbound (red) and inbound (blue) routes; R, right turn; L, left turn. Data from 30 ants that were tracked for 10 consecutive outbound runs and the respective inbound runs. (C) All possible foraging routes from the perspective of the ants, color coded by their route type: asymmetrical, identical and symmetrical. (D) Counts for each of the 16 possible foraging routes.

A 20 cm high plastic barrier encircled the entrance to an ant nest, connected by a short plastic tube (diameter 2 cm) to the sidewall of a 5 m long aluminium channel (7 cm high and wide, with the tube positioned 50 cm away from one end). As a result, every ant leaving the nest entered the channel and had to turn left to reach a feeder, stocked with biscuit crumbs located 4.5 m from the nest entrance. Along this way, the ants encountered two diamond-shaped aluminium sections with central dividers, offering a choice between a left (L) or right (R) passage (Fig. 2A).

Each ant (*n*=30) was individually marked with distinctive dot patterns on its gaster (different enamel colors applied by a needle) and was given approximately 3 h to familiarize itself with the channel and the feeder position during foraging. After this training period, each marked ant was followed for 10 consecutive outbound and inbound runs. At each decision point (left or right), the ant's decision was recorded. For example, if an ant turned L then R on its outbound run and then R and L on its inbound run, it would have completed an LRRL run.

#### Quantification and statistical analysis

To assess whether the frequency of the 16 potentially possible path combinations (Fig. 2C) forms a uniform distribution (where each combination is equally likely), we performed a chi-square goodness-of-fit test.

**Fig. 3.**
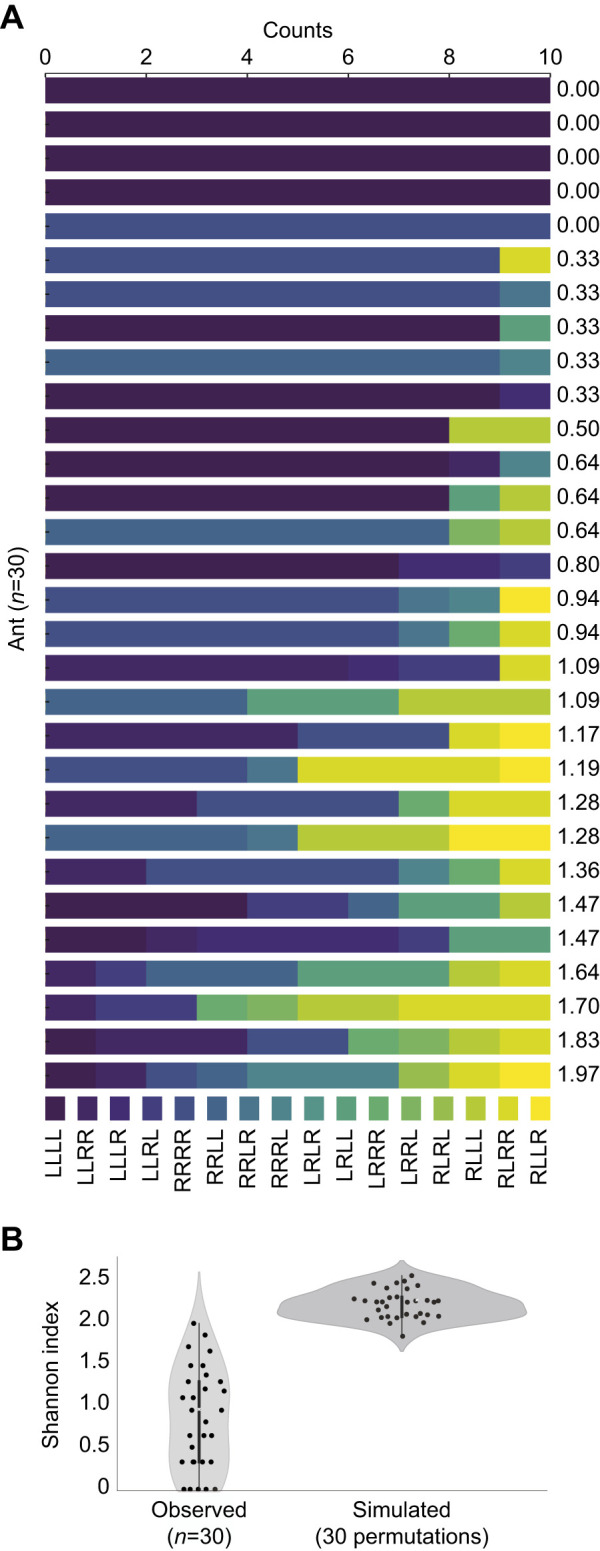
**Desert ants display idiosyncratic decision making.** (A) Distribution of different navigation sequences for each ant. Each bar represents an individual ant, with the different colored segments indicating the frequency of each of the possible routes. The respective Shannon index value for each ant is shown beside each bar. (B) Violin–scatter plots comparing Shannon diversity indices from observed ant behaviors (left) with those from 30 simulations (right) (two-sample *t*-test; *t*=−12.48, *P*<0.001).

Next, to determine whether individual ants showed unique route preferences, we performed a permutation analysis with a thousand synthetic ants that made route decisions randomly. Shannon indices were calculated based on the route combinations among these synthetic ants, ranging from approximately 0.99 to 2.39, with a mean of 1.72. This simulation allowed us to apply the Shannon diversity index, typically used to assess species diversity, to analyze the incidence of specific route combinations (e.g. LRLR, RRRR) among individual ants. We assessed the similarity in navigational behaviors among ants through hierarchical clustering using Ward's method, which minimizes the total within-cluster variance. By calculating Euclidean distances between the navigation paths, we created a dendrogram to visually represent the clusters of navigation behaviors (Fig. 3B). Additionally, we generated a heatmap to visualize the frequency of each decision sequence across ants (Fig. 3B).

## RESULTS AND DISCUSSION

### Idiosyncrasy in an artificially complex environment

The first experiment investigated whether the idiosyncratic navigation observed in *C. fortis* in featureless environments (Buehlmann et al., 2015) extends to more structured settings. An artificial maze was constructed by placing barriers between the ants' nest and a food source. The movement patterns of five individual ants were monitored across five consecutive runs through this maze. Each ant demonstrated a strong preference for specific routes, consistently choosing the same or similar paths during multiple trials. Despite the complexity introduced by the artificial barriers, individual ants exhibited distinct and repeatable navigation choices (Fig. 1A).

Analysis revealed minimal variability in the routes chosen by individual ants across different runs. Most ants had low deviation scores from their initial paths, indicating high route fidelity. The standard deviation of path deviations for each ant was notably low, varying between 0.0 and 0.5 (Fig. 1B). The mean intraindividual variability across all observed ants was 0.2, suggesting a strong tendency to repeat navigation decisions. In contrast, routes chosen by different ants varied significantly. Higher deviation scores were observed when comparing the most common path of each ant against those of their peers, with standard deviations ranging from 1.9 to 2.9 (Fig. 1B). The mean interindividual variability was 2.44, indicating significant differences in the chosen paths. Thus, the intraindividual variability was significantly smaller than the interindividual variability (*t*=−7.697 d.f.=4, *P*=0.002), suggesting that while individual ants are consistent in their navigation choices, considerable variation exists between individuals. We conclude that *C. fortis*, which usually inhabits a flat and featureless environment, like other ants (Barrie et al., 2023; Kohler and Wehner, 2005; Mangan and Webb, 2012; Wystrach et al., 2011b) follows idiosyncratic routes when facing an artificial complex environment.

### Decision-making dynamics in structured environments

Having shown that *C. fortis* navigates along idiosyncratic routes, in a second experiment we investigated the basic rules underlying the formation of such routes. To address this, we created a simplified maze with two visually identical decision points, requiring the ants to choose between turning L or R.

Individual ants were tracked within the maze (Fig. 2A), recording their choices for both outbound and inbound paths during the same foraging bout (for example, outbound LR, inbound LR, resulting in forage bout LRLR; Fig. 2B,C). We subsequently asked whether certain choice combinations occurred more frequently than others. Routes were categorized into three different types: symmetrical routes, asymmetrical routes and identical routes (Fig. 2C). Symmetrical routes involved the same directional choice on both outbound and inbound runs (e.g. RRRR); identical routes had opposite decisions (e.g. LLRR) and asymmetrical routes showed no similarity (e.g. RLLL).

Out of 300 foraging runs made by 30 individuals, 167 were symmetrical, 55 displayed identical routes and only 78 were asymmetrical (Fig. 2C,D).

Notably, over 55% of instances involved a preference for either RRRR or LLLL sequences (Fig. 2D). These frequencies significantly deviated from what would have been expected under equal likelihood, with RRRR and LLLL routes being more common (χ^2^=529.55, d.f.=15, *P*<0.001, Chi-square test). The prevalence of symmetrical routes indicates a consistent choice of either left or right at each decision point during foraging. This consistency suggests that uniform visual cues within the maze lead to repetitive egocentric decision making, regardless of whether the ant is approaching the first or second decision point and irrespective of the direction of travel.

While previous studies have shown that wood ants can retrieve different memories from a single landmark based on their travel direction (Fernandes et al., 2018; Graham and Collett, 2002; Harris et al., 2005), many tested desert ants displayed repeated reactions to a landmark, regardless of the timing or direction of approach. Interestingly, another group of ants showed consistency within inbound and within outbound runs, but varied between them, resulting in identical routes such as LLRR and RRLL (Fig. 2C).

We then asked whether the ants, despite the general preference to perform symmetrical or identical runs, would still follow idiosyncratic routes within the maze. To assess this, we computed the Shannon diversity index, a metric typically used for evaluating species diversity (Shannon, 1948), to determine whether individual route combinations (e.g. LRLR or RRRR) were overrepresented in a single ant, indicating a stable route. With the 16 possible combinations (Fig. 2C,D), the Shannon diversity index could reach values from zero (the ant always took the same route) to 2.77 (the ant always took a different route).

Among the 30 ants followed on 10 consecutive foraging runs, five consistently followed the same path in all trials, another five altered their route only once, while the other ants exhibited different levels of route flexibility (Fig. 3A).

Next, we performed a permutation test using the observed frequencies of different route combinations to determine whether the variability within individual ants was lower than expected from the overall frequencies observed across all ants (Fig. 3B). Our analysis revealed that the observed Shannon indices were significantly lower than those obtained from simulations assuming random decision making (two-sample *t*-test: *t*=−12.48, *P*<0.001). Thus, we conclude that ants consistently follow idiosyncratic routes, even within a simplified maze, with some ants sharing similar routes and others following different ones.

In desert settings, *C. fortis* predominantly relies on its path integrator, whereas other desert ant species from more cluttered environments tend to emphasize learning and using visual landmarks more heavily (Bühlmann et al., 2011; Schwarz and Cheng, 2010). For decades, *C. fortis* and other desert ant species have been studied for their navigational capabilities, both in their natural environment and in artificial mazes (Barrie et al., 2023; Bega et al., 2019, 2020; Kohler and Wehner, 2005; Mangan and Webb, 2012; Saar et al., 2017; Wehner, 2019). This body of work has provided a robust framework for exploring the navigational decision-making processes of *C. fortis*.

Our experiments demonstrated that ants exhibit remarkable consistency in their choices at successive binary decision points, corroborating previous studies on visually driven choices and individual lateralization in ant navigation (Collett, 2010; Frasnelli and Vallortigara, 2018). This consistency suggests fundamental navigational mechanisms may involve view-based learning and motor responses that guide identical choices at visually similar junctions, regardless of whether the ants are on inbound or outbound journeys. Given *Cataglyphis* ants' extensive memory (e.g. Bisch-Knaden and Wehner, 2003; Huber and Knaden, 2018), it is plausible that they can remember multiple decision points along a complex journey, benefiting from consistent handling of similar scenarios.

Although individual ants tend to follow repetitive routes, these routes can vary between individuals. Future experiments would need to control for external factors to determine whether L/R choices are fully individually determined and consistent across more contexts. They should determine whether ants that, for example, prefer L in one type of maze will exhibit a similar preference under different conditions. This could indicate some form of navigational handedness, which might explain the significant overrepresentation of LLLL sequences in our dataset (Fig. 2D). It would also be interesting to investigate whether ants that consistently navigate the same routes belong to specific demographic or experiential groups within the colony's forager force, potentially linking run stability to foraging experience.

Our data show the existence of stable routes when the salt pan-adapted *C. fortis* is experimentally exposed to a complex environment. In using such routes, the ants display a strategy similar to that observed in other desert ants from cluttered environments (Wystrach et al., 2011b).

Our study emphasizes the need for experimental designs that can test specific cognitive rules. By analyzing ant navigation in well-defined structured environments (e.g. mazes, where consecutive decision points are visually identical), we lay the groundwork for future research to isolate the factors influencing navigation decisions. For example, manipulating the visual aspects of junctions or altering setups to assess the impact of prior choices on subsequent decisions could provide concrete evidence of the cognitive processes involved. One could imagine, for example, that making the two decision points in our setup more visually distinct would result in fewer ants that continuously turn either LL or RR during their inbound or outbound runs. Initially, we aimed to identify ants that consistently chose LR or RL patterns in either inbound or outbound routes, as these ants would be ideal candidates for testing additional decision-making rules along idiosyncratic routes. For instance, we wanted to see how an LR ant would have behaved at a second junction if the first one were removed in a test scenario. However, we found that the tendency of ants to repeat the same behavior when encountering the same junction twice was so predominant that it did not allow us to successfully carry out these experiments.

The idiosyncratic navigation patterns observed in *C. fortis* are not unique to ants: similar behaviors have been documented across various taxa, including other insects such as bumblebees and vertebrates such as pigeons and elephants, all of which navigate complex environments using learned and adaptive strategies (Baker, 1978; de Silva and Wittemyer, 2012; Guilford and Biro, 2014; Ohashi et al., 2007). This cross-species prevalence of idiosyncratic navigation accentuates its evolutionary significance and the adaptive advantage in efficiently locating and exploiting resources in dynamic environments.

In conclusion, while our study advances the understanding of *C. fortis*' navigational abilities in structured environments, it also exposes the need for caution in interpreting these results as definitive proof of specific cognitive rules. Rather, our findings should serve as a foundation for more detailed investigations aimed at experimentally dissecting and confirming the cognitive underpinnings of ant navigation. Future studies might explore whether navigational ‘handedness’ or preference for specific directions correlates with other behavioral traits, potentially revealing deeper links between navigation, cognition and ecological success in desert ants.
